# Acute Heat Priming Dampens Gene Expression Response to Thermal Stress in a Widespread *Acropora* Coral

**DOI:** 10.1002/ece3.72938

**Published:** 2026-01-14

**Authors:** Declan J. A. Stick, W. Jason Kennington, Carolina Castro‐Sanguino, Shannon L. Duffy, James P. Gilmour, Luke Thomas

**Affiliations:** ^1^ The UWA Oceans Institute, Indian Ocean Marine Research Centre University of Western Australia Crawley Western Australia Australia; ^2^ The Australian Institute of Marine Science Crawley Western Australia Australia; ^3^ School of Biological Sciences The University of Western Australia Crawley Western Australia Australia; ^4^ Centre for Evolutionary Biology, School of Biological Sciences The University of Western Australia Crawley Western Australia Australia

**Keywords:** coral resilience, gene expression plasticity, Ningaloo Reef, recovery, transcriptional memory, Western Australia

## Abstract

Physiological plasticity is fundamental for resisting environmental change. As climate change accelerates and environmental stressors become more frequent, understanding how habitat‐forming species shift their physiology to match their environment is essential for predicting broader ecosystem responses. In this study, we examined whether prior exposure to sub‐bleaching heat stress influenced the gene expression responses to a subsequent thermal challenge in a common reef‐building coral. We primed *Acropora* corals from the World Heritage‐listed Ningaloo Reef (WHNR) to acute (24 h) sub‐bleaching temperatures (+5°C from the mean monthly maximum MMM, 32°C) before subjecting them to a more intense thermal challenge (+6°C from MMM, 33°C), and assessed the physiological and transcriptional responses in both naïve (no prior preconditioning) and primed corals compared to controls. Both groups mounted large gene expression responses to heat stress (33°C), which returned to baseline after a recovery period (16 h) at control temperatures (27°C, MMM), with no visible signs of physiological stress. However, primed corals showed a dampened stress response relative to naïve corals, marked by a 28% decline in differentially expressed genes and an overall reduction in intensity of expression of those genes compared to controls. Similar patterns were observed in the symbiotic partners, which showed a dampened response within the primed corals compared to the controls, despite no detectable declines in photosynthetic performance within either treatment. Our results show that short‐term preconditioning of corals is associated with transcriptional dampening of key stress response genes, and that corals are capable of rapid transcriptional recovery and resilience to recurrent heat stress.

## Graphical abstract

(a) Experimental design. Corals from 10 genotypes were distributed across two experimental blocks, each containing nine flow‐through tanks. Fragments from five genotypes were placed in each tank. (b) Temperature profiles and sampling time points in the heat stress assay, demonstrating ramp up from control conditions (27ºC, MMM) to the preconditioning treatment (33ºC) and the thermal challenge treatment (34ºC). Naïve treatment is shown in orange, the primed treatment in red and the control in blue. Sampling time points are indicated with dashed vertical lines: ‘T1’ represents the start of the thermal challenge, ‘T2’ represents the end of the heating hold and ‘T3’ represents the end of the recovery period. A single replicate tank of each temperature treatment (containing 5 colony fragments) from each experimental block (*n* = 2) was sampled at each time point.

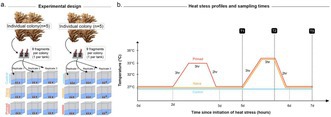

## 1 Introduction

As climate change progresses and severe disturbances become routine, understanding how habitat‐forming species respond to environmental stress is central to predicting the broader response of the ecosystem to climate change. While populations can adapt to changes in their environment across many generations through population‐level shifts in allele frequencies (H. W. Bates 2008; Bonner 1988; Darwin 1859; Grant and Grant 2002), individuals can respond more rapidly through modifications of the phenotype without changes to the underlying DNA sequences (e.g., acclimatisation) (Hofmann and Todgham 2010; López‐Maury et al. 2008; West‐Eberhard 2003). This acclimatisation involves temporary modifications to an organism's physiology, metabolism, or gene expression that enhance survival (López‐Maury et al. 2008). The extent to which organisms can shift their physiology and acclimatise to their environment is central to their ability to survive recurrent stress.

One key mechanism that supports rapid acclimatisation is priming or preconditioning, whereby a mild or sub‐lethal stress exposure prepares an organism for improved performance under subsequent stress (Hilker et al. 2016). Priming has been well documented in terrestrial plants, where controlled exposure to drought, salinity, or heat can enhance crop resilience and yield (Bruce et al. 2007; Hilker et al. 2016; Worrall et al. 2012). Similar responses have been observed in microbial communities, fungi, and some animals, suggesting that priming may be a widespread biological strategy to cope with fluctuating environments (Conrath et al. 2006; Harish and Osherov 2022; Hoffman et al. 2018). Although the molecular mechanisms vary across systems, priming often involves temporary changes in gene expression, metabolism, or epigenetic marks that ‘record’ the initial exposure and enable a faster or altered response upon re‐stimulation—an effect often described as stress memory.

In marine systems, and particularly among reef‐building corals, such mechanisms of stress memory are increasingly recognised as critical to resistance. Coral reefs are dynamic ecosystems periodically subjected to severe disturbances, such as cyclones, outbreaks of predators or diseases, or marine heatwaves (Connell 1997; Cresswell et al. 2024). Many corals already live at the edge of their physiological limits (Burke et al. 2011; Dixon et al. 2022; Sampayo et al. 2016) and have a narrow thermal tolerance range, often just 1°C–2°C above mean maximum temperatures in summer (Glynn 1996; Hoegh‐Guldberg et al. 2007; Hughes et al. 2017). With escalating disturbance regimes associated with anthropogenic climate change, physiological plasticity—including thermal acclimation—becomes critical for coral persistence (Foo and Byrne 2016). Fortunately, corals are capable of increasing thermal thresholds (Barshis et al. 2018; Brown et al. 2002; Castillo et al. 2012; Fisch et al. 2019; Gintert et al. 2018; Hughes et al. 2021; Lough et al. 2018; Palumbi et al. 2014). They can also acclimatise to heat stress by preserving the memory of previous exposures (Hackerott et al. 2021), enabling a more rapid or enhanced response to future stress (Brown and Barott 2022; Hackerott et al. 2021; Hilker et al. 2016; Walter et al. 2013). This stress memory is a complex process, driven by a variety of interrelated mechanisms including shifts in gene expression, microbial symbiosis, and epigenetic modifications (Grottoli et al. 2017; Kenkel and Matz 2016; Palumbi et al. 2014; Putnam 2021; Schoepf et al. 2015).

At the molecular level, the mechanisms underlying thermal priming and stress memory in corals remain poorly understood and appear to vary depending on the duration and intensity of thermal stress (Hackerott et al. 2021; Martell 2023). Priming in coral can involve short‐term transcriptional changes (hours to days), such as transcriptional dampening, where the expression of stress‐response genes—particularly those involved in innate immunity, apoptosis, extracellular matrix formation, and cytoskeletal processes—is reduced under subsequent exposure to heat stress (Bay and Palumbi 2015; Guerrero and Bay 2024). Rather than broadly suppressing stress pathways, acclimation often involves the selective regulation of essential processes, enabling organisms to conserve energy for future function (Rose et al. 2015). Over longer timescales (days to weeks), stress memory can manifest as transcriptional frontloading, where key stress‐response genes maintain elevated baseline expression levels even under non‐stress conditions (Brener‐Raffalli et al. 2022; Vidal‐Dupiol et al. 2022). This frontloading equips organisms to respond more efficiently to repeated stress events, reducing the need for a heightened response when acute stress arises (Barshis et al. 2013; Brener‐Raffalli et al. 2022; Kenkel and Matz 2016; Palumbi et al. 2014).

Despite emerging evidence supporting stress memory and transcriptional plasticity in corals, it is unclear how widespread these mechanisms are across different coral taxa and reef environments. Additionally, transcriptional resilience—a potential determinant of stress tolerance—has received comparatively little attention (Stick et al. 2025), particularly in tracking gene expression dynamics across multiple heat stress events. Addressing these gaps will provide crucial insights into how corals and other sessile organisms persist in an era of climate change and environmental instability.

*Acropora* corals at Ningaloo Reef have exhibited transcriptomic resilience to acute heat stress, rapidly returning gene expression to baseline levels following exposure, with the rate of transcriptional recovery in individual corals linked to the extent of pigment loss (Stick et al. 2025). Building on this finding, we investigate whether a memory of past stress alters the transcriptional response and recovery to subsequent stress. Specifically, we compare the transcriptional responses of both a common *Acropora* and its symbionts in background temperatures (control), when exposed to heat stress (+6°C from MMM, 33°C, for 8 h) without being primed (naïve), and when exposed to heat stress after being primed at (+5°C from MMM, 32°C, for 8 h). By integrating gene expression data with physiological metrics of coral health, we provide insight into the functional groups of genes and mechanisms underlying resilience to recurrent thermal stress and transcriptional recovery. These findings contribute to our broader understanding of how corals and reefs may cope with and adapt to the escalating impacts of climate change.

## 2 Methodology

### 2.1 Study Sites and Sample Collection

Coral samples were collected on 30 November 2023, from Bundegi Reef in the Ningaloo Reef Marine Park, part of the Ningaloo Coast World Heritage Area. Fragments from ten *Acropora* cf. *tenuis* coral colonies were sampled using SCUBA at depths of 3.7 ± 0.6 m along 50–100 m transects and were collected at least 10 m apart from each other to minimise the potential of sampling the same individual twice. Colonies were identified in the field as *Acropora tenuis* based on morphology, following character descriptions provided by Dr. Zoe Richards (Western Australian Museum). However, recent genomic research has revealed two cryptic spawning lineages of this species across offshore reefs in northwestern Australia (Duffy et al. in press; Rosser 2016), with Ningaloo Reef populations found to be dominated exclusively by the autumn‐spawning lineage and associated predominantly with *Cladocopium* symbionts (Duffy et al. in press). In a previous study at the same site, Stick et al. (2025) used genomic data to demonstrate that all sampled colonies belonged to a single cryptic lineage of *Acropora* cf. *tenuis*. Consistent with that finding, SNP‐based clustering analysis conducted in the present study again confirmed that all individuals belonged to a single genetic lineage (see Section 2).

Coral colonies were transported alive in oxygenated tubs with fresh flowing seawater to the Minderoo Foundation Exmouth Research Laboratory (MERL) facility within 1.5 h of collection. Colonies were kept at 26°C ± 0.5°C (ambient site conditions) throughout transport. On arrival to MERL, coral colonies were fragmented into nubbins of ~12 cm^3^ in size and placed into a 1200 L flow‐through acclimation tank. This tank had a recirculation flow of 4000 L/h, programable wavemakers to create optimal flow, and 4× Orphek Atlantic V4 lights that were programmed to low light to avoid added stress (200 μmol photons m^−2^ s^−1^ on a 12:12 h day/night cycle). The acclimatisation system is temperature controlled to an accuracy of up to ±0.5°C and was set to 26°C (ambient site conditions) before being ramped up to the set point of 27°C (control conditions) over 12 h (0.17°C/h). Corals were then left to acclimate for ~24 h. The control temperature was set at 27.0°C ± 1.4°C following in situ 3‐month averages recorded during austral summer (December–February 2022) by (Castro Sanguino et al. 2024).

### 2.2 Heat Stress Assay

**FIGURE 1:**
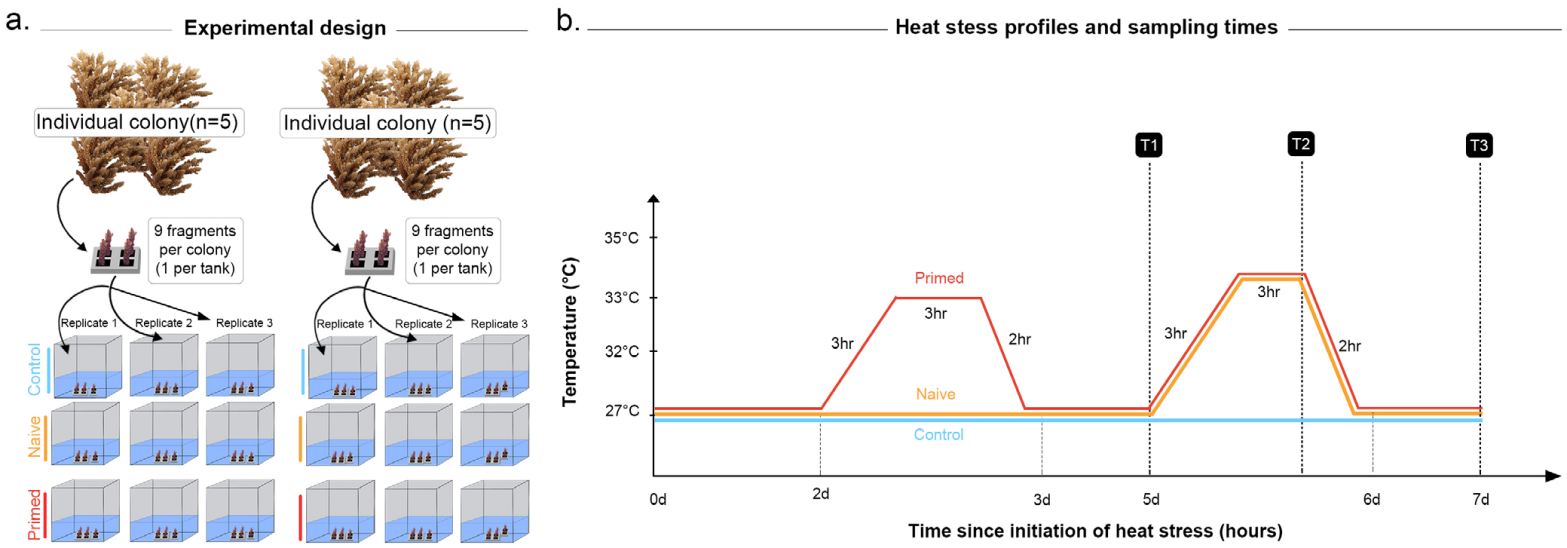
(a) Experimental design, corals from 10 genotypes were distributed across two experimental blocks, each containing nine flow‐through tanks. Fragments from five genotypes were placed in each tank. (b) Temperature profiles and sampling time points in the heat stress assay, demonstrating ramp up from control conditions (27°C, MMM) to the pre‐conditioning treatment (33°C) and the thermal challenge treatment (34°C). Naïve treatment is shown in orange, the primed treatment in red and the control in blue. Sampling time points are indicated with dashed vertical lines: ‘T1’ represents the start of the thermal challenge, ‘T2’ represents the end of the heating hold and ‘T3’ represents the end of the recovery period. A single replicate tank of each temperature treatment (containing 5 colony fragments) from each experimental block (*n* = 2) was sampled at each time point.

Heat stress assays were conducted using the MERL multi‐factor experimental room comprised of 27 × 50 L aquaria, each equipped with 230 V sea water circulators, temperature probes, and Orphek Atlantic V4 light panels. The room has three temperature control systems each with nine tanks, allowing a second three‐level factor to be implemented, while retaining three replicates in each temperature factor combination (i.e., a 3 × 3 × 3 design). All tanks were continuously supplied with local seawater and overhead lights were programmed to mimic Bundegi Reef light conditions (Castro Sanguino et al. 2024) ramping to an average PAR of 528 μmol photons m^−2^ s^−1^ on a 12:12 h day/night cycle. Tanks were split into two experimental blocks each containing nine tanks and a set of five different genotypes (*n* = 10 genotypes in total). The different coral genotypes were dispersed over nine replicated flow‐through tanks in each block by taking fragments from five different coral colonies and placing a fragment from each colony in each replicate tank in each treatment condition (Figure 1a).

Prior experiments determined that bleaching under acute heat stress for these corals occurred at 34°C, and that 33°C was sufficient to produce a large gene expression response (Stick et al. 2025). Based on these data, our experimental design involved exposing a new set of *Acropora* cf. *tenuis* colonies from Bundegi Reef to a preconditioning temperature stress that would elicit a gene expression response, but no physiological stress response (32°C), and a thermal challenge (33°C) known to elicit a much larger gene expression response (Stick et al. 2025). Control samples were kept at 27°C (regional MMM) with no temperature manipulation for the entire 5‐day experiment. The preconditioned tanks were heated to 32°C following an acute heat stress design (Figure 1b), 3 days before being ramped to the thermal challenge treatment (33°C). The preconditioned treatment was held at control levels for 48 h between the preconditioning stress and the thermal challenge. The naïve treatment had no temperature manipulation before being ramped from control conditions to 33°C, with the preconditioned treatment (Figure 1b). Heat treatment profiles were designed following Seneca and Palumbi (2015) and Savary et al. (2021). Temperature ramping started at 10:00 AM, and the heated treatments were gradually increased from the control temperature to the selected temperature treatment over 3 h. The respective temperature was held for 3 h, then decreased back to the control temperature (27°C) over 2 h (6:00 PM). Although CBASS studies often apply longer exposures (e.g., 18 h; Ferrara et al. 2024; Majerova et al. 2021), previous experiments with *Acropora* cf. *tenuis* demonstrated that stress related transcriptomic responses are detectable within 6–8 h at these temperatures (Stick et al. 2025). Therefore, the chosen 8‐h duration for both priming and thermal challenge phases was sufficient to elicit stress responses while minimising the risk of mortality.

To facilitate reproducibility and comparison with other studies, standardised heat stress metrics were calculated using the regional MMM (27°C) as baseline (Voolstra et al. 2025; Nielsen et al. 2022). For the priming phase (32°C; 3 h ramp‐up, 3 h hold, 2 h ramp‐down), the total degree heating hours (DHH) was 27.5°C·h, corresponding to degree heating days (DHD) = 1.15°C·day and degree heating weeks (DHW) = 0.16°C·week, with a ramp rate of 1.67°C·h^−1^. For the thermal challenge (33°C; 3 h ramp‐up, 3 h hold, 2 h ramp‐down), DHH = 33°C·h, DHD = 1.38°C·day, DHW = 0.20°C·week, and ramp rate = 2°C·h^−1^.

A replicate tank for each treatment in each experimental block was selected at random for sampling at each of the three time points: before the start of the thermal challenge (T1–0 h), during the thermal challenge (T2–6 h), and after a period of recovery at control levels (T3–24 h) (Figure 1b). Samples were immediately snap‐frozen in liquid nitrogen at each time point, before being stored at −80°C until further processing.

### 2.3 Laboratory and Bioinformatic Protocols

Total RNA was isolated from 90 coral samples, derived from 10 individuals (five per experimental block), exposed to three temperature treatments (control, naïve and primed), and sampled at three time points (T1, T2 and T3—see Figure 1). For extractions, we used the RNeasy Mini Kit (Qiagen) according to the manufacturer's protocol. Stranded (strand specific) RNAseq libraries were prepared using Agilent's Sureselect HS2 library preparation kit. The protocol includes poly A enrichment followed by iSeq QC, fragmentation, reverse transcription with random primers, ligation with unique molecular barcoded adapters, followed by PCR amplification for indexing. The poly‐A selection in the library preparation reduces unwanted prokaryotic sequences. Library quality control (QC) was performed using Tapestation 4200 and Qubit, followed by QC sequencing on Iseq and Deep sequencing on NovaSeq flow cell at 2 × 150 cycles format to yield approximately 10 million read pairs per sample.

Raw reads were demultiplexed by the sequencing facility. The quality of the demultiplexed reads was checked with FastQC (Andrews 2010) before and after read trimming, and MultiQC (Ewels et al. 2016) was used to concatenate the results of FastQC. Sample sequences were mapped against the coral host transcriptome (30,327 contigs) and the symbiont *Cladocopium* transcriptome (both downloaded from reefgenomics.org) using HISAT2, with a minimum mapping quality of 10. Coral hosts had an average alignment percentage of 91.38% ± 2.38%, while *Symbiodinium* had an average alignment of 53.27% ± 1.73% (for mapping statistics see—File S1). The alignment results were sorted and filtered using SAMtools (Li et al. 2009). The generated sequence alignment map (SAM) was then converted into its binary format, BAM, and count data were extracted for each contig. We normalised our counts matrices with DESeq2 (Love et al. 2014) and contigs with a mean read depth of less than five were excluded from the data set. A total of 17,622 *Acropora* cf. *tenuis* and 17,480 *Symbiodiniaceae* contiguous sequences (contigs) were inferred along with their homologous gene identification codes (IDs) from UniProt (UniProt Consortium 2011), KEGG (Kyoto Encyclopedia of Genes and Genomes), and GO (The Gene Ontology Consortium 2004), using publicly available *Acropora tenuis* gene annotations (downloaded from reefgenomics.org) and *Symbiodinium* gene annotations (downloaded from https://github.com/ckenkel/MontiSymTransgen/blob/master/GeoSymbio_ITS2_LocalDatabase_verForPhyloseq.fasta).

To verify that all coral samples belonged to the same species and to rule out cryptic species‐level divergence, we performed SNP calling and genotype‐based clustering following previously described methods (Stick et al. 2025). A neighbour‐joining tree was constructed in R version 1.79 (R Core Team 2013) using the R package ape (Paradis et al. 2004), based on a Euclidean genetic distance matrix derived from SNP genotypes (File S3: Figure S1). The tree was visualised using ggtree (Yu et al. 2017). All samples clustered together, supporting their classification as a single *Acropora* cf. *tenuis* lineage and confirming that observed transcriptomic differences reflect within‐species (i.e., genotypic) variation.

### 2.4 Differential Expression Analyses

The overall patterns of transcriptional response and recovery were first assessed using principal component analysis (PCA), normalised with variance stabilising transformation (VST) in DeSeq2.0 (Love et al. 2014). VST is calculated from the fitted dispersion‐mean relation(s) and then transforms the count data (normalised by division by the size factors or normalisation factors), yielding a matrix of values that are approximately homoscedastic. PC1 values were separated by timepoint and visualised using density plots, created with ggplot (Wickham 2006). Differential gene expression analyses were also performed with the package DESeq2 (Love et al. 2014). Wald testing for the significant difference of coefficients with a negative binomial general linear model (GLM) was applied in the DESeq function (Love et al. 2014). The analysis was divided based on treatment and timepoint, comparing control conditions with naïve and primed temperatures treatments, as well as comparing the naïve and primed treatments themselves, for all three timepoints. Timepoint and tank effects were evaluated by comparing DEGS between controls and treatments in the two‐block randomised design, accounting for the variation within and between tanks at each timepoint (File S2). DESeq2 *p*‐values were corrected for multiple comparisons using the Benjamini–Hochberg (BH) method. Only the gene contigs with an adjusted *p*‐value less than 0.05 and a log_2_‐fold change of ±2 were considered differently expressed. Differential expression results were visualised using volcano plots, scatter plots and histograms all generated with ggplot2 (Wickham 2006), illustrating log_2_ fold changes, adjusted *p*‐values, and DEG distributions across treatments and timepoints.

### 2.5 Functional Annotations

Gene ontology (GO) was used to highlight the functional differences in the transcriptional response between timepoints and treatments for both host and symbiont. GO enrichment and pathway analyses were performed using the database for annotation, visualisation and integrated discovery (DAVID v6.8). DAVID uses the Fisher's Exact Test to ascertain statistically significant gene enrichment for a particular pathway. Using the functional annotation tool in DAVID, we tested for overrepresentation in our gene modules of GO terms at *p* < 0.05 after Benjamini‐Hochberg correction. Our background list was composed of 17,622 host and 17,480 symbiont contigs that had UniProt annotations, and the gene lists and analysis were separated by treatment and timepoint.

### 2.6 WGCNA Analysis

Weighted Gene Co‐expression Network Analysis (WGCNA) was also performed to identify co‐expression gene modules for sub‐bleaching heat stress. Gene count data were variance‐stabilised using the *vst* function from the DESeq2 R‐package (Love et al. 2014) to normalise expression values across samples. A network was first constructed using all samples across the three treatments (control, naïve, primed). A soft‐thresholding power of 18 was selected based on the scale‐free topology criterion to ensure a scale‐free topology fit. Co‐expression modules were identified using the *blockwiseModules* function in the WGCNA R‐package (Langfelder and Horvath 2008), with a minimum module size of 30 and a merge cut height of 0.25 to combine similar expression patterns. In addition to the combined network, separate WGCNA analyses were conducted for each pairwise treatment comparison (control vs. naïve, control vs. primed, naïve vs. primed) to identify condition‐specific gene co‐expression patterns. For these analyses, a soft‐thresholding power of 10 was chosen based on independent scale‐free topology assessments for each comparison.

Treatment effects on module eigengenes (MEs), which represent the first principal component summarising the expression pattern of a gene co‐expression module, were evaluated using one‐way ANOVA and visualised via boxplots. Correlations between MEs and treatment conditions were calculated and visualised using heatmaps generated with the pheatmap R‐package (Kolde and Kolde 2015). Module sizes and their distribution across comparisons were visualised with dot plots using ggplot2 (Wickham 2006). To characterise the biological relevance of key modules, gene lists from each were functionally annotated using the DAVID Bioinformatics Resource (Huang et al. 2009) based on *Acropora tenuis* gene annotations (downloaded from reefgenomics.org). Functional enrichment analyses were performed to identify overrepresented biological processes, molecular functions, and cellular components associated with each module.

### 2.7 Pigment Quantification and Photosynthetic Performance

We monitored the health of our coral colonies over the course of the experiment using the Coral Health Chart (CHC) and pigment analyses using the photographic method (Siebeck et al. 2006). At timepoints T1: 0 h and T3: 24 h, coral fragments (*n* = 120) were photographed and assigned a colour score using the Coral Health Chart (Siebeck et al. 2006). Digital images of the fragments were taken using an Olympus TG7 camera. For imaging, the mounted fragments, together with a colour reference card and the coral sample ID, were photographed under controlled light conditions and, using the automatic shooting setting in the camera, to minimise light variability between images. The photographic images were then converted into RGB values using Adobe Photoshop CC (Adobe Systems Incorporated, San Jose, CA, USA) based on NEF (RAW) files. The coral apical branch surface was selected using the ‘Quick Selection tool’, avoiding the tip and injured area where nubbins were fragmented, and the average colour values were found using the function Filter > Blur > Average. This produced the colour values used to visually compare to the Coral Health Chart.

Pulse Amplitude Modulate (PAM) fluorometry was also used to assess coral symbiont (Symbiodiniaceae) health after heat‐stress via measures of *F*_v_/*F*_m_ which represents the ratio of the variable fluorescence (*F*_v_) to the maximum fluorescence (*F*_m_) where *F*_v_ is the difference between minimum (*F*_o_) and maximum fluorescence (*F*_m_) during a saturating pulse. *F*_v_/*F*_m_ measurements were taken before and after the thermal priming, and before (T1) and after the triggering heat stress (T3) following a 2–3 h dark‐acclimation period (Figure 1b). *F*_v_/*F*_m_ measurements were taken following a 2‐ to 3‐h period of dark‐acclimation after the initial preconditional stress (T1), and after the heat‐stress challenge (T3) (Figure 1b) using a Diving‐PAM underwater fluorometer (Walz) with settings: light intensity = 8, light frequency = 3, electronic gain = 2, damping = 2. Two *F*_v_/*F*_m_ measurements were taken at a constant distance of ~2 mm from the coral tissue of each nubbin to account for any spatial variability in the photochemical response (Fitt et al. 2001), but avoiding the tip and injured areas where nubbins were fragmented.

A linear mixed‐effects model, implemented using the *lmer* function from the R package lme4 (D. M. Bates 2010), was used to assess variation in coral pigmentation loss (CHC scores—continuous variables) and symbiont health (*F*_v_/*F*_m_) across temperature treatments at each timepoint. The model included ‘temperature treatment’ and ‘block’ as fixed effects to account for systematic differences between experimental blocks, and ‘individual’ (genotype) and ‘tank’ as random effects to account for variability within coral genotypes and across tanks (CHC or *F*_v_/*F*_m_ ~ treatment + block + (1|genotype) + (1|tank)). The significance of the fixed effect was assessed using a Wald's Chi square test with the Anova function from the car R‐package (Fox et al. 2007). Post hoc pairwise comparisons of treatment means were performed using the *emmeans* and *pairs* functions from the emmeans R‐package (Lenth 2016). Tukey's method was applied for multiple testing corrections to identify significant differences between treatment levels. The estimated marginal means (EMMs) for the treatment variable were also obtained. Although residuals were not normally distributed for CHC scores, visual inspection of residual plots indicated that deviations from normality were mild and acceptable for LMM analysis, and models are robust to violations of assumption (Schielzeth et al. 2020). Permutation test results were consistent with the model presented, showing no significant difference for either CHC or *F*_v_/*F*_m_ when controlling for block (File S3).

## 3 Results

### 3.1 Large but Transient Transcriptional Response to Sub‐Bleaching Heat Stress

**FIGURE 2:**
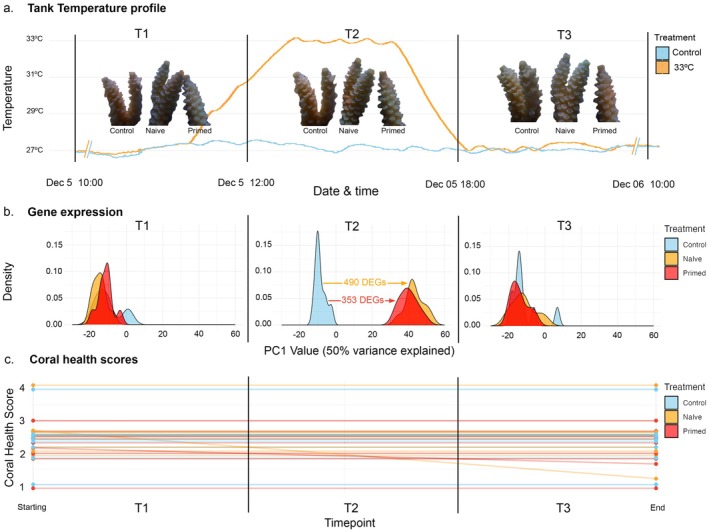
(a) Tank temperature profiles for control (MMM, 27°C) and heat stress (+6°C, 33°C) treatments. (b) Density plot of PC1 values for transcriptome‐wide gene expression of *Acropora* cf. *tenuis* at the beginning of the thermal challenge (T1), 6 h into short‐term heat stress (T2), and 24 h post (T3) short‐term heat stress. Colours represent each temperature treatment and control. Differentially expressed genes (DEGs) between treatment and controls are shown at T2. (c) Coral health scores of individuals in each treatment from the beginning of the thermal challenge (T1) to the end of the experiment (T3).

In all corals, we identified a pronounced gene expression response to heat stress (+6°C from MMM, 33°C), despite no other physiological signs of stress (Figure 2a,b). Indeed, both pigment analyses of the coral host (Coral Health Scores) and photochemical response (*F*_v_/*F*_m_) of the symbiont showed no detectable changes in our control, naïve, or primed treatments during the priming exposure or the subsequent thermal challenge, suggesting a strong disconnect between the genetic stress response and two commonly used metrics of physiological stress in corals and symbionts (Figure 2c, File S3: Tables S1 and S2). The large transcriptomic responses to heat stress were largely transient, and all corals showed a nearly complete return to baseline levels of gene expression 16 h after returning to control temperatures (T3) (Figure 2b). At T3, almost all (> 98%) of DEGs had returned to baseline expression levels in all treatments (Figure 2b, File S2).

### 3.2 Primed Corals Show a Dampened Response During Stress (T2)

**FIGURE 3:**
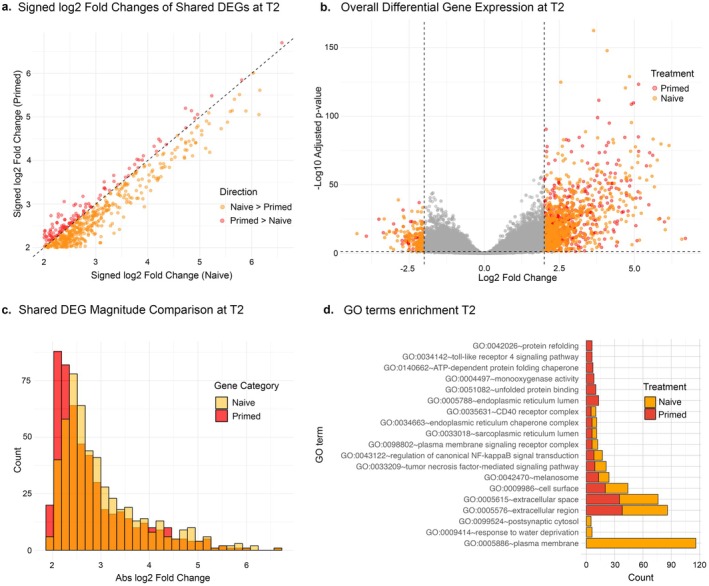
(a) Scatterplot comparing signed log_2_ fold changes of DEGs shared between treatments. Points above the dashed diagonal line indicate genes more strongly upregulated in primed samples, while those below indicate stronger upregulation in naïve samples. (b) Volcano plot displaying log_2_ fold change versus –log_10_ adjusted *p*‐value (*p*_adj_) for differential gene expression between control versus naïve and control versus primed comparisons. Genes meeting the significance threshold (*p*_adj_ ≤ 0.05 and |log_2_FC| ≥ 2) are highlighted in orange (naïve) and red (primed); all other genes are shown in grey. Dashed vertical lines indicate log_2_FC cutoffs of ±2, and the horizontal line marks the significance threshold of *p*_adj_ = 0.05. (c) Overlaid histogram of absolute log_2_ fold changes for shared DEGs, highlighting the distribution of expression magnitudes between treatments. (d) Bar plot of top enriched gene ontology terms for comparisons of heat stress treatments 6 h into the thermal challenge; naïve and primed treatments are colour‐coded. All terms were enriched with a maximum FDR‐adjusted *p*‐value of 0.05.

Despite both treatments mounting a large transcriptomic response that recovered 24 h later, primed corals exhibited a smaller overall transcriptional response relative to naïve corals, with a 28% reduction in the number of differentially expressed genes (DEGs) during heat stress (File S2). Six hours into the thermal challenge (T2), naïve individuals mounted a gene expression response that involved 490 DEGs, the majority of which (~82%) were upregulated (Figure 3a,b, File S2). These genes showed strong homology to functions associated with the plasma membrane, extracellular space, and immune signalling, including components of the tumour necrosis factor signalling pathway, canonical NF‐kappaB signal transduction, and the CD40 receptor complex (File S2, Figure 3d). CD40 is known to activate NF‐κB signalling (Hostager and Bishop 2013), suggesting that the upregulation of CD40‐related genes may directly contribute to the transcriptional activation of downstream immune and stress‐response pathways in corals during heat stress. In contrast, the corals primed before heat stress had only 353 DEGs identified at T2 compared to controls (Figure 3a, File S2). Notably, there was a substantial overlap (97%) in the DEGs identified in the naïve and primed treatments, indicating a shared response to thermal stress.

In addition, the magnitude of gene expression change was consistently lower in primed corals compared to naïve corals. Among the shared DEGs, 79% (259 DEGs) showed reduced log_2_ fold changes in the primed treatment compared to the naïve treatment (Figure 3a). This trend was reflected in the overall DEG distribution, with primed corals displaying both a lower number and smaller change in expression of individual genes than those within the naïve treatment (Figure 3b). Additionally, primed individuals showed a lower frequency of genes with large‐magnitude fold changes compared to naïve corals, suggesting a less intense transcriptional shift (Figure 3c).

Beyond the shared transcriptional response to heat stress, differences also emerged in the identity of uniquely expressed genes and their associated functions. An entirely new set of genes was unique to the naïve treatment (148 DEGs), compared to the 11 unique DEGs in the primed treatment (File S2). Although the primed treatment shared many Gene Ontology (GO) terms with the naïve treatment, it also displayed distinct terms related to protein homeostasis, such as protein refolding, unfolded protein binding, ATP‐dependent protein folding chaperones, and endoplasmic reticulum lumen (Figure 3d). Interestingly, the primed treatment also showed significant enrichment in the toll‐like receptor 4 signalling pathway, monooxygenase activity, and extracellular space—categories not observed in the naïve treatment (Figure 3d). In contrast, the naïve group exhibited significant enrichment for postsynaptic cytosol and plasma membrane (Figure 3d). Notably, most (89%) genes within these functional categories were upregulated during heat stress (Figure 2b, File S2).

The dampened response observed at T2 in primed corals is unlikely to be due to frontloading. Prior to heating (T1), no differences in gene expression (DEGs) were observed between primed and naïve corals, nor between primed individuals and controls (Figure 2b, File S2). In fact, the data from T1 demonstrate that all individuals had returned to baseline levels of gene expression, with no upregulation of DEGs heading into the experimental heat stress (+6 from MMM, 33°C) after the initial stress priming (+5 from MMM, 32°C) (Figure 2b, File S2). This suggests that the dampened response at T2 in primed corals results from a mechanism other than frontloading.

### 3.3 Few Gene Networks of Large Effect Show Signs of Dampening in Primed Corals

**FIGURE 4:**
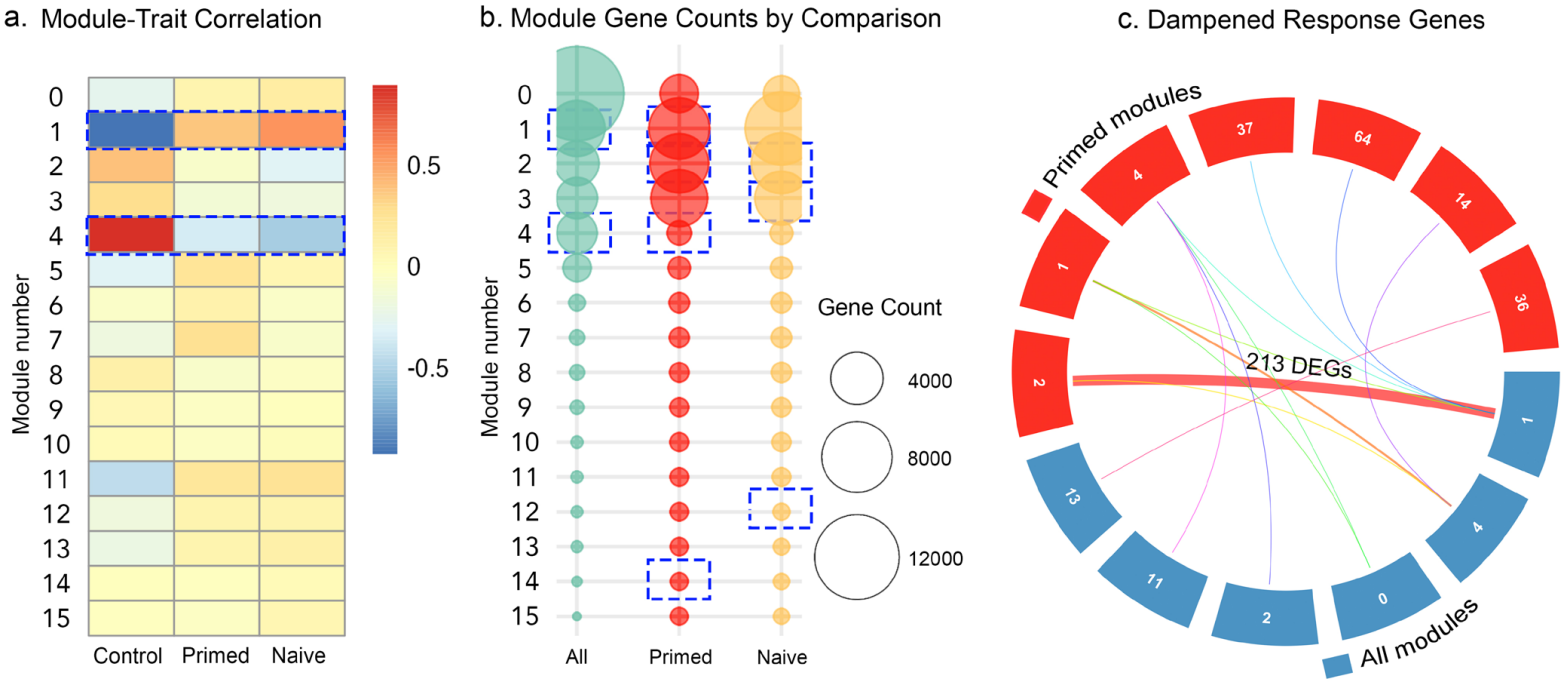
(a) Heatmap of module–trait correlations for all samples. Each cell shows the Pearson correlation coefficient between module eigengenes and experimental conditions (Control, Naïve, Primed). Colour intensity indicates correlation strength, with red representing positive and blue representing negative correlations. (b) Dot plot showing the number of genes assigned to each WGCNA module across different pairwise comparisons. Dot size reflects the total number of genes in each module for the corresponding comparison. Module significance is indicated in each figure (blue dashed outline). For clarity, only the first 15 modules are shown for the primed and naïve comparisons, although additional significant modules exist. (c) Circos plot showing overlaps between co‐expression modules from primed samples and the full dataset, specifically for genes with a dampened expression response. Sectors represent modules; link thickness and colour reflect the number and grouping of shared dampened genes.

The transcriptional response to heat stress in *Acropora* cf. *tenuis* was organised into 15 co‐expression modules (all treatments analysed together), ranging in size from 37 to 4955 contigs (Figure 4a, File S5). Modules 1 and 4 captured the majority of the stress response and were significantly different across control, naïve, and primed treatments (Figure 4a,b). Module 1 could not be functionally characterised overall, but it comprised 4955 genes, including 1023 annotated with protein binding functions, suggesting a broad role in protein interactions (File S4). In contrast, Module 4 was associated with costamere structure and function (File S5). Post hoc analyses revealed that Modules 1 and 4 were significantly different between control and both heat‐treated groups, but not between naïve and primed corals (File S3: Tables S3 and S4).

When treatment comparisons were analysed independently (e.g., control vs. primed), similar patterns emerged. The comparison between control and naïve corals identified 9 significantly affected modules, with two of those (Modules 2 and 3) showing larger gene counts (Figure 4b, File S5). However, neither of these modules could be functionally annotated through enrichment analysis. A direct comparison between control and primed corals identified 16 significantly affected modules, including three of larger size—Modules 1, 2, and 4 (Figure 4b, File S5). Among these, only Module 2 could be functionally enriched, showing a significant association with endoplasmic reticulum (ER) processes (File S5).

Interestingly, of the 269 dampened DEGs detected—which represent 79% of the total primed DEGs—the majority clustered within module 2 (the ER response module identified in the primed pairwise comparison) or module 1 (from the overall comparison) (Figure 4c). This suggests that the ER response observed in primed corals is encompassed within the broader stress‐related module 1.

While no modules were significantly different between primed and naïve corals, the module–trait correlation heatmap (Figure 4a) suggested a more muted transcriptional response in primed corals. For example, Module 1 showed a stronger correlation with the naïve group (*r* = 0.55) compared to primed (*r* = 0.38), and Module 4 followed a similar trend (*r* = −0.53 for naïve vs. –0.36 for primed). These weaker correlations imply a less coordinated activation of key stress‐related modules in primed corals, consistent with a dampened overall response to heat exposure.

### 3.4 Symbionts Also Show Signs of Stress Memory

Symbiont gene expression mirrored patterns observed in the host, with a substantial number of genes expressed at T2, followed by near complete recovery to baseline levels by T3 (File S6). However, the overall response in symbionts was markedly less pronounced compared to the host. Differential expression analysis revealed 202 genes differentially expressed between control and naïve groups, and 164 genes differentially expressed between control and primed groups (File S6). The majority of these genes were upregulated in both cases. However, unlike the host, Gene Ontology (GO) enrichment analysis did not reveal common functional categories among the symbiont DEGs.

## 4 Discussion

We combined transcriptome‐wide gene expression analyses with physiological metrics of coral health to explore the role of thermal preconditioning on coral stress responses and recovery. Both coral hosts and their symbionts primed to sub‐bleaching heat stress temperatures (32°C) exhibited a markedly dampened transcriptional response to a subsequent acute heat stress at 33°C, consistent with a form of stress memory. Compared to naïve corals, primed corals showed a reduced magnitude of expression across several heat stress–associated genes and core regulatory networks during the second thermal exposure, highlighting transcriptional dampening as a key feature of the recurrent stress response. Both naïve and primed corals exhibited near‐complete recovery of gene expression profiles following sub‐bleaching heat exposure, indicating an inherent capacity to mount a large and dynamic response to stress and restore it to maintain basic metabolic functions once it has passed. Our findings add to the growing understanding of the role of gene expression plasticity and preconditioning to coral resilience and highlight key mechanisms and functional groups involved, with broader implications for coral conservation in the face of ongoing climate change.

### 4.1 Transcriptional Resilience

The ability of organisms to mount and subsequently recover a gene expression response to stress is critical for their long‐term survival and fitness, particularly in fluctuating environments (De Nadal et al. 2011; Rivera et al. 2021). In the present study, we show that *Acropora* species from parts of Ningaloo Reef mount a large and coordinated transcriptional response when exposed to sub‐bleaching heat stress. Remarkably, this response subsides rapidly once the thermal stress is removed, with expression profiles returning to baseline levels. This rapid transcriptional recovery suggests a pronounced physiological capacity for disturbance absorption and homeostatic regulation—a key feature of resilience in *Acropora* corals from the Ningaloo Reef (Stick et al. 2025).

This capacity to dynamically adjust gene expression in response to environmental fluctuations is not unique to corals. Similar transcriptional plasticity has been documented in intertidal mussels (Gracey et al. 2008), which experience regular thermal and desiccation stress and show rapid induction and repression of heat shock proteins to cope with tidal cycles. Likewise, in the filamentous red alga *Bangia* sp., heat stress memory has been shown to differentially regulate the expression of nitrogen transporter genes, with transcripts displaying attenuated re‐induction during repeated heat exposure (Sato et al. 2024). Across these diverse taxa, the ability to mount large, but transient, transcriptional responses appears to be a critical strategy for organisms inhabiting variable or extreme environments.

In corals, this resilience may reflect evolutionary tuning to highly variable reef environments where frequent but sub‐lethal stress events are common (Brener‐Raffalli et al. 2022; Brown et al. 2025).

### 4.2 Transcriptional Dampening as a Result of Stress Memory

Stress priming occurs when prior exposure to stress alters organisms' responses to future stressors and is widely observed in corals that survive repeated bleaching events, often resulting in increased thermal tolerance (Brown and Barott 2022; Ferrara et al. 2024; Hackerott et al. 2021; Martell 2023). Unlike constitutive changes in stress‐related gene expression observed in some heat‐tolerant corals (Barshis et al. 2013; Brener‐Raffalli et al. 2022; Fifer et al. 2021; Vidal‐Dupiol et al. 2022), primed corals in our study exhibited a targeted dampening of stress‐related gene expression upon subsequent heat exposure, indicative of stress memory. This selective transcriptional adjustment likely prioritises essential cellular functions and conserves energy by reducing the activation of large‐scale, energetically costly stress responses. Supporting this interpretation, Han et al. (2024) showed that heat shock protein (HSP) expression declined during prolonged stress across multiple coral species, suggesting that maintaining elevated HSP levels is metabolically demanding and may be actively downregulated as an energy‐saving strategy. Similarly, Bay and Palumbi (2017) found that corals with dampened expression of metabolic and ribosomal processing genes under stress demonstrated greater thermal tolerance, implying that repression of non‐essential transcriptional programs can enhance resilience. Together, these findings emphasise that transcriptional plasticity, not just constitutive expression, is central to coral resilience and that dampening may represent a regulated, adaptive mechanism to minimise energetic costs and preserve cellular integrity in increasingly hotter and variable ocean conditions (Bay and Palumbi 2015, 2017; Han et al. 2024; Rivera et al. 2021).

While transcriptional dampening could be seen as an acclimatisation strategy for conserving energy, another possibility is that primed corals simply lack the energetic capacity to mount another large‐scale response after the initial priming stress (e.g., a metabolic trade‐off). If this is the case, it becomes a matter of balancing the maintenance of homeostasis with the capacity to acclimatise to new environmental stressors. This interpretation aligns with the broader hypothesis of ‘energy limitation’, where an organism's capacity to respond to stress becomes constrained by its metabolic resources (Guppy and Withers 1999; Sokolova 2013).

However, the nature of the dampened transcriptional response observed in this study strongly supports the interpretation of stress memory rather than energy depletion. Specifically, the dampened expression in primed corals was not indicative of system‐wide suppression. Instead, it occurred in a targeted and coordinated fashion, especially within key stress‐associated gene groups and modules, such as those involved in endoplasmic reticulum function (Barshis et al. 2013; Maor‐Landaw and Levy 2016; Ron and Walter 2007). This pattern suggests deliberate regulatory adjustment, linked to memory‐based modulation of the stress response, rather than physiological exhaustion. Furthermore, physiological indicators such as the *F*_v_/*F*_m_ photochemical response and the visual health scores based on tissue colour remained stable across treatments and throughout the duration of the experiment, indicating that corals maintained sufficient physiological capacity to mount a second stress response and that the coral host response may act to buffer other components of the coral holobiont from sub‐optimal conditions. Taken together, these transcriptional and physiological patterns support a model in which prior thermal exposure enables corals to recalibrate their stress response, allowing for a more efficient and less energetically costly response upon subsequent stress. This is consistent with a growing body of evidence that corals acclimated to elevated temperatures exhibit a dampened gene expression response to stress, highlighting the role of stress memory as a mechanism underpinning resilience (Bay and Palumbi 2015; Grottoli et al. 2014; Han et al. 2024).

### 4.3 Gene Co‐Expression Networks Provide Further Evidence for Transcriptional Dampening in Primed Corals

The identification of 15 co‐expression modules reveals a highly structured and coordinated transcriptional architecture underpinning the coral stress response. Among these, two co‐expression modules captured the majority of the transcriptional changes during heat stress. These modules were significantly different between control, naïve, and primed treatments, but not between naïve and primed corals. This suggests that heat stress does not alter the regulation of all gene networks in *Acropora* cf. *tenuis*, but rather only a select few that are linked to stress response. In other words, a relatively small number of large, functionally enriched modules drive the stress response, and priming acts to dampen their activation rather than recruit entirely new regulatory programs. Stress adaptation is often governed by a limited number of core regulatory networks, rather than a broad‐scale transcriptomic response, a pattern consistent with observations in other model systems. For example, in *Arabidopsis thaliana* , drought and heat stress lead to the reactivation of core stress‐responsive transcriptional modules, but with altered intensity depending on prior exposure, suggesting a memory‐like modulation of network dynamics (Lämke and Bäurle 2017). Similarly, in yeast, repeated stress exposures selectively reshape expression levels within conserved co‐expression modules, rather than inducing novel (Berry and Gasch 2008; Gasch et al. 2000). These parallels highlight that resilience can be achieved by modulating the strength, not the structure, of existing gene regulatory networks in response to environmental history.

Moreover, in our study, when each treatment comparison was examined separately, only a handful of modules showed significant differences—further underscoring the focused nature of the response. The main difference unique to the primed treatment was Module 2, with a strong endoplasmic reticulum (ER) signature—unsurprising given this pathway was also highlighted in both the GO enrichment and DEG analyses. The enrichment of endoplasmic reticulum (ER)‐associated genes in Module 2, particularly in primed corals, may point to a central role for ER function in managing cellular stress and facilitating recovery. The ER is critical for protein folding, calcium storage, and the unfolded protein response (UPR)—a key pathway activated during thermal stress when protein homeostasis is disrupted (Hetz 2012; Ron and Walter 2007). Sustained or severe heat stress can overwhelm the ER's capacity, leading to proteotoxic stress and cell damage (Barshis et al. 2013; Ron and Walter 2007). In primed corals, the enhanced expression of ER‐related genes may reflect a state of preparedness, enabling more efficient handling of misfolded proteins and restoration of protein homeostasis during subsequent stress events (Maor‐Landaw and Levy 2016). This is consistent with a protective, anticipatory role of ER function in stress memory, where prior exposure tunes cellular machinery to respond more effectively to future insults. Given its cross‐talk with apoptosis, redox regulation, and calcium signalling, modulation of ER activity may be a key component of the coral's ability to tolerate and recover from heat stress.

### 4.4 Protein Homeostasis as a Key Mechanism of Resilience to Heat Stress

When comparing the gene ontology (GO) terms from this study to those in our previous study (Stick et al. 2025), 53% were the same. Both studies identified protein refolding and unfolded protein binding as important functional categories. In our previous study, these terms were significantly associated with coral bleaching. Similarly, in the current study, primed corals showed a downregulation of these categories. This suggests and adds to previous findings that the management of protein homeostasis plays a crucial role in preventing bleaching, emphasising the importance of regulating these pathways in response to thermal stress (Barshis et al. 2013; Seneca and Palumbi 2015).

While Stick et al. (2025) identified immune signalling pathways, such as tumour necrosis factor‐mediated signalling pathway and NF‐kappaB regulation, the current study also highlighted additional terms related to protein homeostasis, like ATP‐dependent protein folding chaperone and endoplasmic reticulum lumen, that were more prominent in primed corals. These distinctions suggest differences in the transcriptional response between the two studies, potentially driven by the experimental priming treatment.

### 4.5 Symbiont Responses: Gene Expression and Photochemical Efficiency

Symbiont gene expression analysis revealed significant transcriptional responses to heat stress that paralleled those of the coral host—primed corals exhibited reduced symbiont gene expression changes compared to naïve counterparts. However, the overall magnitude of transcriptional responses in symbionts was much lower than in the host. Despite significant changes in symbiont gene expression, there was no corresponding shift in the photochemical response (*F*_v_/*F*_m_) between treatment groups. While *F*_v_/*F*_m_ is widely used to assess photosynthetic performance, it may not capture subcellular stress or longer‐term impacts on symbiont function. Previous studies have also reported gene expression changes or pigment loss occurred in the absence of detectable changes in *F*_v_/*F*_m_ (Gomez‐Campo et al. 2022; Jones and Hoegh‐Guldberg 2001; Roth 2014). Similarly, research on the coral *Oculina arbuscula* and its symbiont *Breviolum psygmophilum* showed that symbionts in hospite exhibited a dampened transcriptional response to thermal stress compared to those ex hospite, suggesting that the host microenvironment can buffer symbionts against environmental stressors (Aichelman et al. 2024). Nonetheless, the observed transcriptional dampening in our coral symbionts indicates that symbionts possess intrinsic buffering mechanisms, but to a lesser extent than the host, and that their recovery is strongly shaped by the host's ability to regulate stress.

### 4.6 Implications for Coral Conservation and Future Research

Our study highlights the capacity of corals from the Ningaloo Reef to withstand acute thermal stress, demonstrating both transcriptional resilience and a form of transcriptional ‘memory’. This ability to mount a rapid and transient response to repeated heat exposure may be a crucial factor underpinning coral survival in an era of accelerating climate change. It is also important to understand whether an acute response translates into long‐term resilience in the face of more frequent, prolonged, and intense heat stress events.

Indeed, we must critically examine whether thermal priming has real potential as a conservation tool or whether its value lies more in advancing our understanding of the mechanisms behind coral acclimatisation. Future research should investigate the duration of stress memory, the thresholds beyond which repeated exposures become detrimental, and the ecological relevance of priming in wild populations. If the benefits of priming diminish with overly frequent stress events, as suggested by previous work (Crisp et al. 2016; Hackerott et al. 2021; Hughes et al. 2019), then coral resilience strategies must balance the timing, intensity, and frequency of thermal exposures. Ultimately, a deeper understanding of stress memory dynamics will be vital to informing conservation approaches that aim to enhance coral resilience in our rapidly warming oceans.

## Author Contributions

**Declan J. A. Stick:** conceptualization (lead), data curation (lead), formal analysis (lead), funding acquisition (lead), investigation (lead), methodology (lead), project administration (lead), validation (lead), visualization (lead), writing – original draft (lead), writing – review and editing (lead). **W. Jason Kennington:** conceptualization (supporting), data curation (supporting), formal analysis (supporting), funding acquisition (supporting), investigation (equal), methodology (supporting), project administration (equal), resources (equal), supervision (equal), validation (equal), visualization (equal), writing – original draft (equal), writing – review and editing (equal). **Carolina Castro‐Sanguino:** data curation (supporting), investigation (supporting), methodology (supporting), supervision (supporting), validation (supporting), writing – original draft (supporting), writing – review and editing (supporting). **Shannon L. Duffy:** data curation (supporting), investigation (supporting), methodology (supporting), validation (supporting), writing – original draft (supporting), writing – review and editing (supporting). **James P. Gilmour:** validation (supporting), writing – original draft (supporting), writing – review and editing (equal). **Luke Thomas:** conceptualization (equal), data curation (supporting), formal analysis (equal), funding acquisition (supporting), investigation (equal), methodology (equal), project administration (equal), resources (equal), supervision (equal), validation (equal), visualization (equal), writing – original draft (equal), writing – review and editing (equal).

## Funding

Funding for this work was provided by the Keiran McNamara World Heritage PhD Top‐Up Scholarship to D.J.A.S. This research is supported by the Australian Institute of Marine Science (AIMS) under its AIMS‐Minderoo Coral Resilience Project.

## Disclosure

Benefit‐sharing statement: Benefits from this research were generated via collaboration between students and scientists from the Australian Institute of Marine Science and the University of Western Australia. Moreover, corals from the Ningaloo Reef are a conservation priority, and results found herein are shared with the broader scientific community.

## Ethics Statement

This work did not require ethical approval from an animal welfare committee. All coral was collected under DBCA permit FO25000401.

## Conflicts of Interest

The authors declare no conflicts of interest.

## Supporting information


**File S1:** ece372938‐sup‐0001‐SupplementaryFile1.xlsx.

**File S2:** ece372938‐sup‐0002‐SupplementaryFile2.xlsx.

**File S3:** ece372938‐sup‐0003‐SupplementaryFile3.docx.

**File S4:** ece372938‐sup‐0004‐SupplementaryFile4.xlsx.

**File S5:** ece372938‐sup‐0005‐SupplementaryFile5.xlsx.

**File S6:** ece372938‐sup‐0006‐SupplementaryFile6.xlsx.

## Data Availability

The raw RNA sequencing data and metadata have been deposited in the National Centre for Biotechnology Information (NCBI) Sequence Read Archive (SRA) database under accession number PRJNA1307646. All other data, including scripts, are available from the Dryad data repository https://doi.org/10.5061/dryad.vq83bk46k.

## References

[ece372938-bib-0001] Aichelman, H. E. , A. K. Huzar , D. M. Wuitchik , et al. 2024. “Symbiosis Modulates Gene Expression of Symbionts, but Not Coral Hosts, Under Thermal Challenge.” Molecular Ecology 33, no. 8: e17318. 10.1111/mec.17318. 38488669

[ece372938-bib-0002] Andrews, S. 2010. “FastQC: A Quality Control Tool for High Throughput Sequence Data.” Babraham Bioinformatics, Cambridge, United Kingdom.

[ece372938-bib-0003] Barshis, D. J. , C. Birkeland , R. J. Toonen , R. D. Gates , and J. H. Stillman . 2018. “High‐Frequency Temperature Variability Mirrors Fixed Differences in Thermal Limits of the Massive Coral *Porites lobata* .” Journal of Experimental Biology 221, no. 24: jeb188581. 30305375 10.1242/jeb.188581

[ece372938-bib-0004] Barshis, D. J. , J. T. Ladner , T. A. Oliver , F. O. Seneca , N. Traylor‐Knowles , and S. R. Palumbi . 2013. “Genomic Basis for Coral Resilience to Climate Change.” Proceedings of the National Academy of Sciences 110, no. 4: 1387–1392. 10.1073/pnas.1210224110. PMC3557039 23297204

[ece372938-bib-0005] Bates, D. M. 2010. lme4: Mixed‐Effects Modeling With R. Springer.

[ece372938-bib-0006] Bates, H. W. 2008. “Contributions to an Insect Fauna of the Amazon Valley (Lepidoptera: Heliconidae).” Biological Journal of the Linnean Society 16, no. 1: 41–54. 10.1111/j.1095-8312.1981.tb01842.x.

[ece372938-bib-0007] Bay, R. A. , and S. R. Palumbi . 2015. “Rapid Acclimation Ability Mediated by Transcriptome Changes in Reef‐Building Corals.” Genome Biology and Evolution 7, no. 6: 1602–1612. 25979751 10.1093/gbe/evv085 PMC4494073

[ece372938-bib-0008] Bay, R. A. , and S. R. Palumbi . 2017. “Transcriptome Predictors of Coral Survival and Growth in a Highly Variable Environment.” Ecology and Evolution 7, no. 13: 4794–4803. 28690808 10.1002/ece3.2685 PMC5496549

[ece372938-bib-0009] Berry, D. B. , and A. P. Gasch . 2008. “Stress‐Activated Genomic Expression Changes Serve a Preparative Role for Impending Stress in Yeast.” Molecular Biology of the Cell 19, no. 11: 4580–4587. 18753408 10.1091/mbc.E07-07-0680 PMC2575158

[ece372938-bib-0010] Bonner, J. T. 1988. The Evolution of Complexity by Means of Natural Selection. Princeton University Press.

[ece372938-bib-0011] Brener‐Raffalli, K. , J. Vidal‐Dupiol , M. Adjeroud , et al. 2022. “Gene Expression Plasticity and Frontloading Promote Thermotolerance in Pocillopora Corals.” Peer Community Journal 2: e13.

[ece372938-bib-0012] Brown, B. , C. Downs , R. Dunne , and S. Gibb . 2002. “Exploring the Basis of Thermotolerance in the Reef Coral *Goniastrea aspera* .” Marine Ecology Progress Series 242: 119–129.

[ece372938-bib-0013] Brown, K. T. , and K. L. Barott . 2022. “The Costs and Benefits of Environmental Memory for Reef‐Building Corals Coping With Recurring Marine Heatwaves.” Integrative and Comparative Biology 62, no. 6: 1748–1755. 35661887 10.1093/icb/icac074

[ece372938-bib-0014] Brown, K. T. , Z. Dellaert , M. P. Martynek , et al. 2025. “Extreme Environmental Variability Induces Frontloading of Coral Biomineralisation Genes to Maintain Calcification Under pCO(2) Variability.” Molecular Ecology 34, no. 2: e17603. 10.1111/mec.17603. 39605240 PMC11701869

[ece372938-bib-0015] Bruce, T. J. , M. C. Matthes , J. A. Napier , and J. A. Pickett . 2007. “Stressful ‘Memories’ of Plants: Evidence and Possible Mechanisms.” Plant Science 173, no. 6: 603–608.

[ece372938-bib-0016] Burke, L. , K. Reytar , M. Spalding , and A. Perry . 2011. Reefs at Risk Revisited. World Resources Institute (WRI).

[ece372938-bib-0017] Castillo, K. D. , J. B. Ries , J. M. Weiss , and F. P. Lima . 2012. “Decline of Forereef Corals in Response to Recent Warming Linked to History of Thermal Exposure.” Nature Climate Change 2, no. 10: 756–760.

[ece372938-bib-0018] Castro Sanguino, C. , D. Stick , S. Duffy , C. Grimaldi , J. Gilmour , and L. Thomas . 2024. “Differential Impacts of Light on Coral Phenotypic Responses to Acute Heat Stress.” Journal of Experimental Marine Biology and Ecology 581: 152057. 10.1016/j.jembe.2024.152057.

[ece372938-bib-0019] Connell, J. H. 1997. “Disturbance and Recovery of Coral Assemblages.” Coral Reefs 16: S101–S113.

[ece372938-bib-0020] Conrath, U. , G. J. Beckers , V. Flors , et al. 2006. “Priming: Getting Ready for Battle.” Molecular Plant‐Microbe Interactions 19, no. 10: 1062–1071. 17022170 10.1094/MPMI-19-1062

[ece372938-bib-0021] Cresswell, A. K. , M. Renton , T. J. Langlois , D. P. Thomson , J. Lynn , and J. Claudet . 2024. “Coral Reef State Influences Resilience to Acute Climate‐Mediated Disturbances.” Global Ecology and Biogeography 33, no. 1: 4–16.

[ece372938-bib-0022] Crisp, P. A. , D. Ganguly , S. R. Eichten , J. O. Borevitz , and B. J. Pogson . 2016. “Reconsidering Plant Memory: Intersections Between Stress Recovery, RNA Turnover, and Epigenetics.” Science Advances 2, no. 2: e1501340. 10.1126/sciadv.1501340. 26989783 PMC4788475

[ece372938-bib-0023] Darwin, C. 1859. On the Origin of Species by Means of Natural Selection. John Murray.

[ece372938-bib-0024] De Nadal, E. , G. Ammerer , and F. Posas . 2011. “Controlling Gene Expression in Response to Stress.” Nature Reviews Genetics 12, no. 12: 833–845. 10.1038/nrg3055 22048664

[ece372938-bib-0025] Dixon, A. M. , P. M. Forster , S. F. Heron , A. M. Stoner , and M. Beger . 2022. “Future Loss of Local‐Scale Thermal Refugia in Coral Reef Ecosystems.” PLOS Climate 1, no. 2: e0000004.

[ece372938-bib-0095] Duffy, S. , W. J. Kennington , C. Castro‐Sanguino , et al. in press. “Patterns of Genetic Connectivity, Symbiont Associations, and Acute Heat Tolerance in a Broadcast Spawning Coral Along a Fringing Reef in Western Australia.” Journal of Ecology and Evolution.

[ece372938-bib-0027] Ewels, P. , M. Magnusson , S. Lundin , and M. Käller . 2016. “MultiQC: Summarize Analysis Results for Multiple Tools and Samples in a Single Report.” Bioinformatics 32, no. 19: 3047–3048. 27312411 10.1093/bioinformatics/btw354 PMC5039924

[ece372938-bib-0028] Ferrara, E. F. , A. Roik , F. Woehrmann‐Zipf , and M. Ziegler . 2024. “Thermal Preconditioning Modulates Coral Physiology and Heat Tolerance: A Multi‐Species Perspective.” *bioRxiv*. 10.1101/2024.07.18.604102. PMC12060272 40279456

[ece372938-bib-0029] Fifer, J. , B. Bentlage , S. Lemer , A. G. Fujimura , M. Sweet , and L. J. Raymundo . 2021. “Going With the Flow: How Corals in High‐Flow Environments Can Beat the Heat.” Molecular Ecology 30, no. 9: 2009–2024. 33655552 10.1111/mec.15869

[ece372938-bib-0030] Fisch, J. , C. Drury , E. K. Towle , R. N. Winter , and M. W. Miller . 2019. “Physiological and Reproductive Repercussions of Consecutive Summer Bleaching Events of the Threatened Caribbean Coral *Orbicella faveolata* .” Coral Reefs 38: 863–876.

[ece372938-bib-0031] Fitt, W. K. , B. E. Brown , M. E. Warner , and R. P. Dunne . 2001. “Coral Bleaching: Interpretation of Thermal Tolerance Limits and Thermal Thresholds in Tropical Corals.” Coral Reefs 20: 51–65.

[ece372938-bib-0032] Foo, S. , and M. Byrne . 2016. “Acclimatization and Adaptive Capacity of Marine Species in a Changing Ocean.” Advances in Marine Biology 74: 69–116. 27573050 10.1016/bs.amb.2016.06.001

[ece372938-bib-0033] Fox, J. , G. G. Friendly , S. Graves , et al. 2007. The Car Package. Vol. 1109. R Foundation for Statistical Computing.

[ece372938-bib-0034] Gasch, A. P. , P. T. Spellman , C. M. Kao , et al. 2000. “Genomic Expression Programs in the Response of Yeast Cells to Environmental Changes.” Molecular Biology of the Cell 11, no. 12: 4241–4257. 11102521 10.1091/mbc.11.12.4241 PMC15070

[ece372938-bib-0096] Gene Ontology Consortium . 2004. “The Gene Ontology (GO) Database and Informatics Resource.” Nucleic Acids Research 32, no. 90001: 258D–261D. 10.1093/nar/gkh036. PMC308770 14681407

[ece372938-bib-0035] Gintert, B. E. , D. P. Manzello , I. C. Enochs , et al. 2018. “Marked Annual Coral Bleaching Resilience of an Inshore Patch Reef in the Florida Keys: A Nugget of Hope, Aberrance, or Last Man Standing?” Coral Reefs 37: 533–547.

[ece372938-bib-0036] Glynn, P. W. 1996. “Coral Reef Bleaching: Facts, Hypotheses and Implications.” Global Change Biology 2, no. 6: 495–509.

[ece372938-bib-0037] Gomez‐Campo, K. , S. Enríquez , and R. Iglesias‐Prieto . 2022. “A Road Map for the Development of the Bleached Coral Phenotype.” Frontiers in Marine Science 9: 806491. 10.3389/fmars.2022.806491.

[ece372938-bib-0038] Gracey, A. Y. , M. L. Chaney , J. P. Boomhower , W. R. Tyburczy , K. Connor , and G. N. Somero . 2008. “Rhythms of Gene Expression in a Fluctuating Intertidal Environment.” Current Biology 18, no. 19: 1501–1507. 10.1016/j.cub.2008.08.049. 18848447

[ece372938-bib-0039] Grant, P. R. , and B. R. Grant . 2002. “Unpredictable Evolution in a 30‐Year Study of Darwin's Finches.” Science 296, no. 5568: 707–711. 11976447 10.1126/science.1070315

[ece372938-bib-0040] Grottoli, A. G. , D. Tchernov , and G. Winters . 2017. “Physiological and Biogeochemical Responses of Super‐Corals to Thermal Stress From the Northern Gulf of Aqaba, Red Sea.” Frontiers in Marine Science 4: 215.

[ece372938-bib-0098] Grottoli, A. G. , M. E. Warner , S. J. Levas , et al. 2014. “The Cumulative Impact of Annual Coral Bleaching can Turn Some Coral Species Winners Into Losers.” Global Change Biology 20, no. 12: 3823–3833. 10.1111/gcb.12658. 25044878

[ece372938-bib-0041] Guerrero, L. , and R. Bay . 2024. “Patterns of Methylation and Transcriptional Plasticity During Thermal Acclimation in a Reef‐Building Coral.” Evolutionary Applications 17: e13757. 10.1111/eva.13757. 39027686 PMC11254580

[ece372938-bib-0042] Guppy, M. , and P. Withers . 1999. “Metabolic Depression in Animals: Physiological Perspectives and Biochemical Generalizations.” Biological Reviews 74, no. 1: 1–40. 10396183 10.1017/s0006323198005258

[ece372938-bib-0043] Hackerott, S. , H. A. Martell , and J. M. Eirin‐Lopez . 2021. “Coral Environmental Memory: Causes, Mechanisms, and Consequences for Future Reefs.” Trends in Ecology & Evolution 36, no. 11: 1011–1023. 34366170 10.1016/j.tree.2021.06.014

[ece372938-bib-0044] Han, T. , X. Liao , Z. Guo , J.‐Y. Chen , C. He , and Z. Lu . 2024. “Deciphering Temporal Gene Expression Dynamics in Multiple Coral Species Exposed to Heat Stress: Implications for Predicting Resilience.” Science of the Total Environment 912: 169021. 38061659 10.1016/j.scitotenv.2023.169021

[ece372938-bib-0045] Harish, E. , and N. Osherov . 2022. “Fungal Priming: Prepare or Perish.” Journal of Fungi 8, no. 5: 448. 35628704 10.3390/jof8050448 PMC9145559

[ece372938-bib-0046] Hetz, C. 2012. “The Unfolded Protein Response: Controlling Cell Fate Decisions Under ER Stress and Beyond.” Nature Reviews Molecular Cell Biology 13, no. 2: 89–102. 22251901 10.1038/nrm3270

[ece372938-bib-0047] Hilker, M. , J. Schwachtje , M. Baier , et al. 2016. “Priming and Memory of Stress Responses in Organisms Lacking a Nervous System.” Biological Reviews 91, no. 4: 1118–1133. 10.1111/brv.12215. 26289992

[ece372938-bib-0048] Hoegh‐Guldberg, O. , P. J. Mumby , A. J. Hooten , et al. 2007. “Coral Reefs Under Rapid Climate Change and Ocean Acidification.” Science 318, no. 5857: 1737–1742. 18079392 10.1126/science.1152509

[ece372938-bib-0049] Hoffman, A. J. , J. W. Finger Jr. , and H. Wada . 2018. “Early Stress Priming and the Effects on Fitness‐Related Traits Following an Adult Stress Exposure.” Journal of Experimental Zoology Part A: Ecological and Integrative Physiology 329, no. 6–7: 323–330. 10.1002/jez.2190. 29896887

[ece372938-bib-0050] Hofmann, G. E. , and A. E. Todgham . 2010. “Living in the Now: Physiological Mechanisms to Tolerate a Rapidly Changing Environment.” Annual Review of Physiology 72: 127–145. 10.1146/annurev-physiol-021909-135900. 20148670

[ece372938-bib-0051] Hostager, B. S. , and G. A. Bishop . 2013. “CD40‐Mediated Activation of the NF‐κB2 Pathway.” Frontiers in Immunology 4: 376. 24298274 10.3389/fimmu.2013.00376 PMC3828524

[ece372938-bib-0052] Huang, D. W. , B. T. Sherman , and R. A. Lempicki . 2009. “Systematic and Integrative Analysis of Large Gene Lists Using DAVID Bioinformatics Resources.” Nature Protocols 4, no. 1: 44–57. 19131956 10.1038/nprot.2008.211

[ece372938-bib-0053] Hughes, T. P. , M. L. Barnes , D. R. Bellwood , et al. 2017. “Coral Reefs in the Anthropocene.” Nature 546, no. 7656: 82–90. 10.1038/nature22901. 28569801

[ece372938-bib-0055] Hughes, T. P. , J. T. Kerry , S. R. Connolly , et al. 2019. “Ecological Memory Modifies the Cumulative Impact of Recurrent Climate Extremes.” Nature Climate Change 9, no. 1: 40–43.

[ece372938-bib-0054] Hughes, T. P. , J. T. Kerry , S. R. Connolly , et al. 2021. “Emergent Properties in the Responses of Tropical Corals to Recurrent Climate Extremes.” Current Biology 31, no. 23: 5393–5399. 34739821 10.1016/j.cub.2021.10.046

[ece372938-bib-0056] Jones, R. J. , and O. Hoegh‐Guldberg . 2001. “Diurnal Changes in the Photochemical Efficiency of the Symbiotic Dinoflagellates (*Dinophyceae*) of Corals: Photoprotection, Photoinactivation and the Relationship to Coral Bleaching.” Plant, Cell & Environment 24, no. 1: 89–99. 10.1046/j.1365-3040.2001.00648.x.

[ece372938-bib-0057] Kenkel, C. D. , and M. V. Matz . 2016. “Gene Expression Plasticity as a Mechanism of Coral Adaptation to a Variable Environment.” Nature Ecology & Evolution 1, no. 1: 1–6. 10.1038/s41559-016-0014 28812568

[ece372938-bib-0058] Kolde, R. , and M. R. Kolde . 2015. Package ‘pheatmap’. R Package, 1(7). CRAN.

[ece372938-bib-0059] Lämke, J. , and I. Bäurle . 2017. “Epigenetic and Chromatin‐Based Mechanisms in Environmental Stress Adaptation and Stress Memory in Plants.” Genome Biology 18, no. 1: 124. 28655328 10.1186/s13059-017-1263-6 PMC5488299

[ece372938-bib-0060] Langfelder, P. , and S. Horvath . 2008. “WGCNA: An R Package for Weighted Correlation Network Analysis.” BMC Bioinformatics 9, no. 1: 1–13. 19114008 10.1186/1471-2105-9-559 PMC2631488

[ece372938-bib-0061] Lenth, R. V. 2016. “Least‐Squares Means: The R Package Lsmeans.” Journal of Statistical Software 69: 1–33.

[ece372938-bib-0062] Li, H. , B. Handsaker , A. Wysoker , et al. 2009. “The Sequence Alignment/Map Format and SAMtools.” Bioinformatics 25, no. 16: 2078–2079. 19505943 10.1093/bioinformatics/btp352 PMC2723002

[ece372938-bib-0063] López‐Maury, L. , S. Marguerat , and J. Bähler . 2008. “Tuning Gene Expression to Changing Environments: From Rapid Responses to Evolutionary Adaptation.” Nature Reviews Genetics 9, no. 8: 583–593. 10.1038/nrg2398 18591982

[ece372938-bib-0064] Lough, J. M. , K. D. Anderson , and T. P. Hughes . 2018. “Increasing Thermal Stress for Tropical Coral Reefs: 1871–2017.” Scientific Reports 8, no. 1: 6079. 10.1038/s41598-018-24530-9. 29666437 PMC5904187

[ece372938-bib-0065] Love, M. , S. Anders , and W. Huber . 2014. “Differential Analysis of Count Data—The DESeq2 Package.” Genome Biology 15: 550. 10.1186/s13059-014-0550-8. 25516281 PMC4302049

[ece372938-bib-0094] Magrane, M. , and UniProt Consortium . 2011. “UniProt Knowledgebase: A Hub of Integrated Protein Data.” Database 2011: bar009. 10.1093/database/bar009. 21447597 PMC3070428

[ece372938-bib-0091] Majerova, E. , F. C. Carey , C. Drury , and R. D. Gates . 2021. “Preconditioning Improves Bleaching Tolerance in the Reef‐Building Coral *Pocillopora acuta* Through Modulations in the Programmed Cell Death Pathways.” Molecular Ecology 30, no. 14: 3560–3574. 10.1111/mec.15988. 34008873

[ece372938-bib-0066] Maor‐Landaw, K. , and O. Levy . 2016. “Gene Expression Profiles During Short‐Term Heat Stress; Branching vs. Massive Scleractinian Corals of the Red Sea.” PeerJ 4: e1814. 10.7717/peerj.1814. 27069783 PMC4824894

[ece372938-bib-0067] Martell, H. A. 2023. “Thermal Priming and Bleaching Hormesis in the Staghorn Coral, *Acropora cervicornis* (Lamarck 1816).” Journal of Experimental Marine Biology and Ecology 560: 151820. 10.1016/j.jembe.2022.151820.

[ece372938-bib-0093] Nielsen, J. J. V. , G. Matthews , K. R. Frith , et al. 2022. “Experimental Considerations of Acute Heat Stress Assays to Quantify Coral Thermal Tolerance.” Scientific Reports 12, no. 1: 16831. 10.1038/s41598-022-20138-2. 36207307 PMC9546840

[ece372938-bib-0068] Palumbi, S. R. , D. J. Barshis , N. Traylor‐Knowles , and R. A. Bay . 2014. “Mechanisms of Reef Coral Resistance to Future Climate Change.” Science 344, no. 6186: 895–898. 24762535 10.1126/science.1251336

[ece372938-bib-0097] Paradis, E. , J. Claude , and K. Strimmer . 2004. “APE: Analyses of Phylogenetics and Evolution in R language.” Bioinformatics 20, no. 2: 289–290. 10.1093/bioinformatics/btg412. 14734327

[ece372938-bib-0069] Putnam, H. M. 2021. “Avenues of Reef‐Building Coral Acclimatization in Response to Rapid Environmental Change.” Journal of Experimental Biology 224, no. S1: jeb239319. 33627470 10.1242/jeb.239319

[ece372938-bib-0070] R Core Team . 2013. R: A Language and Environment for Statistical Computing. R Foundation for Statistical Computing.

[ece372938-bib-0071] Rivera, H. E. , H. E. Aichelman , J. E. Fifer , et al. 2021. “A Framework for Understanding Gene Expression Plasticity and Its Influence on Stress Tolerance.” Molecular Ecology 30, no. 6: 1381–1397. 10.1111/mec.15820. 33503298

[ece372938-bib-0072] Ron, D. , and P. Walter . 2007. “Signal Integration in the Endoplasmic Reticulum Unfolded Protein Response.” Nature Reviews Molecular Cell Biology 8, no. 7: 519–529. 17565364 10.1038/nrm2199

[ece372938-bib-0073] Rose, N. H. , F. O. Seneca , and S. R. Palumbi . 2015. “Gene Networks in the Wild: Identifying Transcriptional Modules That Mediate Coral Resistance to Experimental Heat Stress.” Genome Biology and Evolution 8, no. 1: 243–252. 10.1093/gbe/evv258. 26710855 PMC4758253

[ece372938-bib-0074] Rosser, N. L. 2016. “Demographic History and Asynchronous Spawning Shape Genetic Differentiation Among Populations of the Hard Coral *Acropora tenuis* in Western Australia.” Molecular Phylogenetics and Evolution 98: 89–96. 10.1016/j.ympev.2016.02.004. 26876640

[ece372938-bib-0075] Roth, M. 2014. “The Engine of the Reef: Photobiology of the Coral–Algal Symbiosis.” Frontiers in Microbiology 5: 422. 25202301 10.3389/fmicb.2014.00422 PMC4141621

[ece372938-bib-0076] Sampayo, E. M. , T. Ridgway , L. Franceschinis , G. Roff , O. Hoegh‐Guldberg , and S. Dove . 2016. “Coral Symbioses Under Prolonged Environmental Change: Living Near Tolerance Range Limits.” Scientific Reports 6, no. 1: 36271. 10.1038/srep36271. 27805069 PMC5090243

[ece372938-bib-0077] Sato, N. , H. V. Khoa , and K. Mikami . 2024. “Heat Stress Memory Differentially Regulates the Expression of Nitrogen Transporter Genes in the Filamentous Red Alga ‘*Bangia*’ sp. ESS1.” Frontiers in Plant Science 15: 1331496. 38375079 10.3389/fpls.2024.1331496 PMC10875135

[ece372938-bib-0090] Savary, R. , D. J. Barshis , C. R. Voolstra , et al. 2021. “Fast and Pervasive Transcriptomic Resilience and Acclimation of Extremely Heat‐Tolerant Coral Holobionts From the Northern Red Sea.” Proceedings of the National Academy of Sciences of the United States of America 118, no. 19: e2023298118. 10.1073/pnas.2023298118. 33941698 PMC8126839

[ece372938-bib-0078] Schielzeth, H. , N. J. Dingemanse , S. Nakagawa , et al. 2020. “Robustness of Linear Mixed‐Effects Models to Violations of Distributional Assumptions.” Methods in Ecology and Evolution 11, no. 9: 1141–1152. 10.1111/2041-210X.13434.

[ece372938-bib-0079] Schoepf, V. , A. G. Grottoli , S. J. Levas , et al. 2015. “Annual Coral Bleaching and the Long‐Term Recovery Capacity of Coral.” Proceedings of the Royal Society B: Biological Sciences 282, no. 1819: 20151887. 10.1098/rspb.2015.1887. PMC4685810 26582020

[ece372938-bib-0080] Seneca, F. O. , and S. R. Palumbi . 2015. “The Role of Transcriptome Resilience in Resistance of Corals to Bleaching.” Molecular Ecology 24, no. 7: 1467–1484. 10.1111/mec.13125. 25728233

[ece372938-bib-0081] Siebeck, U. , N. Marshall , A. Klüter , and O. Hoegh‐Guldberg . 2006. “Monitoring Coral Bleaching Using a Colour Reference Card.” Coral Reefs 25, no. 3: 453–460.

[ece372938-bib-0082] Sokolova, I. M. 2013. “Energy‐Limited Tolerance to Stress as a Conceptual Framework to Integrate the Effects of Multiple Stressors.” Integrative and Comparative Biology 53, no. 4: 597–608. 10.1093/icb/ict028. 23615362

[ece372938-bib-0083] Stick, D. , W. J. Kennington , C. Castro Sanguino , et al. 2025. “Transcriptomic Resilience to Heat Stress in a Wide‐Spread *Acropora* Coral.” Coral Reefs 44, no. 5: 1535–1548. 10.1007/s00338-025-02722-w.

[ece372938-bib-0084] Vidal‐Dupiol, J. , E. Harscouet , D. Shefy , et al. 2022. “Frontloading of Stress Response Genes Enhances Robustness to Environmental Change in Chimeric Corals.” BMC Biology 20, no. 1: 167. 35879753 10.1186/s12915-022-01371-7 PMC9316358

[ece372938-bib-0092] Voolstra, C. R. , R. Alderdice , L. Colin , S. Staab , A. Apprill , and J.‐B. Raina . 2025. “Standardized Methods to Assess the Impacts of Thermal Stress on Coral Reef Marine Life.” Annual Review of Marine Science 17, no. 1: 193–226. 10.1146/annurev-marine-032223-024511. 39116436

[ece372938-bib-0085] Walter, J. , A. Jentsch , C. Beierkuhnlein , and J. Kreyling . 2013. “Ecological Stress Memory and Cross Stress Tolerance in Plants in the Face of Climate Extremes.” Environmental and Experimental Botany 94: 3–8.

[ece372938-bib-0086] West‐Eberhard, M. 2003. Developmental Plasticity and Evolution. Vol. 816. Oxford University Press.

[ece372938-bib-0087] Wickham, H. 2006. “An Introduction to ggplot: An Implementation of the Grammar of Graphics in R.” Statistics 1: 1–8.

[ece372938-bib-0088] Worrall, D. , G. H. Holroyd , J. P. Moore , et al. 2012. “Treating Seeds With Activators of Plant Defence Generates Long‐Lasting Priming of Resistance to Pests and Pathogens.” New Phytologist 193, no. 3: 770–778. 22142268 10.1111/j.1469-8137.2011.03987.x

[ece372938-bib-0089] Yu, G. , D. K. Smith , H. Zhu , Y. Guan , and T. T. Y. Lam . 2017. “Ggtree: An R Package for Visualization and Annotation of Phylogenetic Trees With Their Covariates and Other Associated Data.” Methods in Ecology and Evolution 8, no. 1: 28–36.

