# Supplementary material for: Acute Heat Priming Dampens Gene Expression Response to Thermal Stress in a Widespread Acropora Coral
Source: Ecol Evol. 2026 Jan 14;16(1):e72938. doi: 10.1002/ece3.72938 (PMC12800923; doi:10.1002/ece3.72938)
Supplement: Supplementary file 3 — File S3: ece372938‐sup‐0003‐SupplementaryFile3.docx. [file ECE3-16-e72938-s002.docx]

Supplementary File 3

**Table S1:** ANOVA results and post-hoc comparisons for coral health scores (CHC) across temperature treatments.

The table presents results from the linear mixed-effects model (lmer) assessing the effect of temperature treatments on coral health (CHC) scores, with random effects for Block, Genotype, and Tank. The ANOVA tests the significance of fixed effects (Treatment) and random effects. Residuals were checked for normality using a Shapiro-Wilk test. Post-hoc pairwise comparisons of treatment means were performed using the emmeans package, with Tukey’s method applied for multiple testing corrections. The results show no significant differences between treatment levels for CHC. Additionally, permutation tests were performed ( p value = 0.711 with 1000 permutations).

| Analysis of variance table | npar | Sum sq | Mean Sq | F value |  |
| --- | --- | --- | --- | --- | --- |
| Treatment | 2 | 0.116 | 0.058 | 0.699 |  |
|  |  |  |  |  |  |
| Contrast | Estimate | SE | df | t.ratio | p.value |
| Control - Naive | -0.15 | 0.129 | 2 | -1.162 | 0.5741 |
| Control - Primed | -0.05 | 0.129 | 2 | -0.387 | 0.9237 |
| Naive - Primed | 0.1 | 0.129 | 2 | 0.775 | 0.7518 |

p.value <- lmer.perm.test(CHC, 1000) = 0.97

**Table S2:** ANOVA results and post-hoc comparisons for photosynthetic efficiency (fv/fm) across temperature treatments.

The table presents results from the linear mixed-effects model (lmer) assessing the effect of temperature treatments on coral health (CHC) scores, with random effects for Block, Genotype, and Tank. The ANOVA tests the significance of fixed effects (Treatment) and random effects. Residuals were checked for normality using a Shapiro-Wilk test. Post-hoc pairwise comparisons of treatment means were performed using the emmeans package, with Tukey’s method applied for multiple testing corrections. The results show no significant differences between treatment levels for fv/fm. Additionally, permutation tests were performed (p value = 0.47 with 1000 permutations).

| Analysis of variance table | npar | Sum sq | Mean Sq | F value |  |
| --- | --- | --- | --- | --- | --- |
| Treatment | 2 | 0.0005 | 0.0003 | 0.963 |  |
|  |  |  |  |  |  |
| Contrast | Estimate | SE | df | t.ratio | p.value |
| Control - Naive | -0.009 | 0.00731 | 2 | -1.17 | 0.5707 |
| Control - Primed | 0.0005 | 0.00731 | 2 | 0.062 | 0.998 |
| Naive - Primed | 0.009 | 0.00731 | 2 | 1.231 | 0.546 |

p.value <- lmer.perm.test(fv/fm, 1000) = 0.558

**Table S3:** ANOVA results testing the effect of Treatment on Module Eigengene ME1, including Tukey’s Honest Significant Difference (HSD) post-hoc test for Module ME1 showing pairwise comparisons between Treatment groups.

| Source | Df | Sum Sq | Mean Sq | F value | Pr(>F) |
| --- | --- | --- | --- | --- | --- |
| Treatment | 2 | 0.8679 | 0.434 | 78.87 | 2.81 × 10⁻¹¹ *** |
| Residuals | 24 | 0.1321 | 0.0055 |  |  |

*Significance codes: 0 ‘***’ 0.001 ‘**’ 0.01 ‘*’ 0.05 ‘.’ 0.1 ‘ ’ 1*

**Tukey Multiple Comparisons of Means (95% family-wise confidence level)**

| Comparison | diff | Lower CI | Upper CI | Adjusted p-value |
| --- | --- | --- | --- | --- |
| NaIve - Control | 0.4017 | 0.3144 | 0.489 | < 0.0001 |
| Primed - Control | 0.3546 | 0.2672 | 0.4419 | < 0.0001 |
| Primed - NaIve | -0.0472 | -0.1345 | 0.0402 | 0.3830 |

**Table S4:** ANOVA results testing the effect of Treatment on Module Eigengene ME4, including Tukey’s Honest Significant Difference (HSD) post-hoc test for Module ME4 showing pairwise comparisons between Treatment groups.

| Source | Df | Sum Sq | Mean Sq | F value | Pr(>F) |
| --- | --- | --- | --- | --- | --- |
| Treatment | 2 | 0.8108 | 0.4054 | 51.42 | 2.11 × 10⁻⁹ *** |
| Residuals | 24 | 0.1892 | 0.0079 |  |  |

*Significance codes: 0 ‘***’ 0.001 ‘**’ 0.01 ‘*’ 0.05 ‘.’ 0.1 ‘ ’ 1*

**Tukey Multiple Comparisons of Means (95% family-wise confidence level)**

| Comparison | diff | Lower CI | Upper CI | Adjusted p-value |
| --- | --- | --- | --- | --- |
| NaIve - Control | -0.3888 | -0.4933 | -0.2843 | < 0.0001 |
| Primed - Control | -0.3419 | -0.4465 | -0.2374 | < 0.0001 |
| Primed - NaIve | 0.0469 | -0.0577 | 0.1514 | 0.5116 |


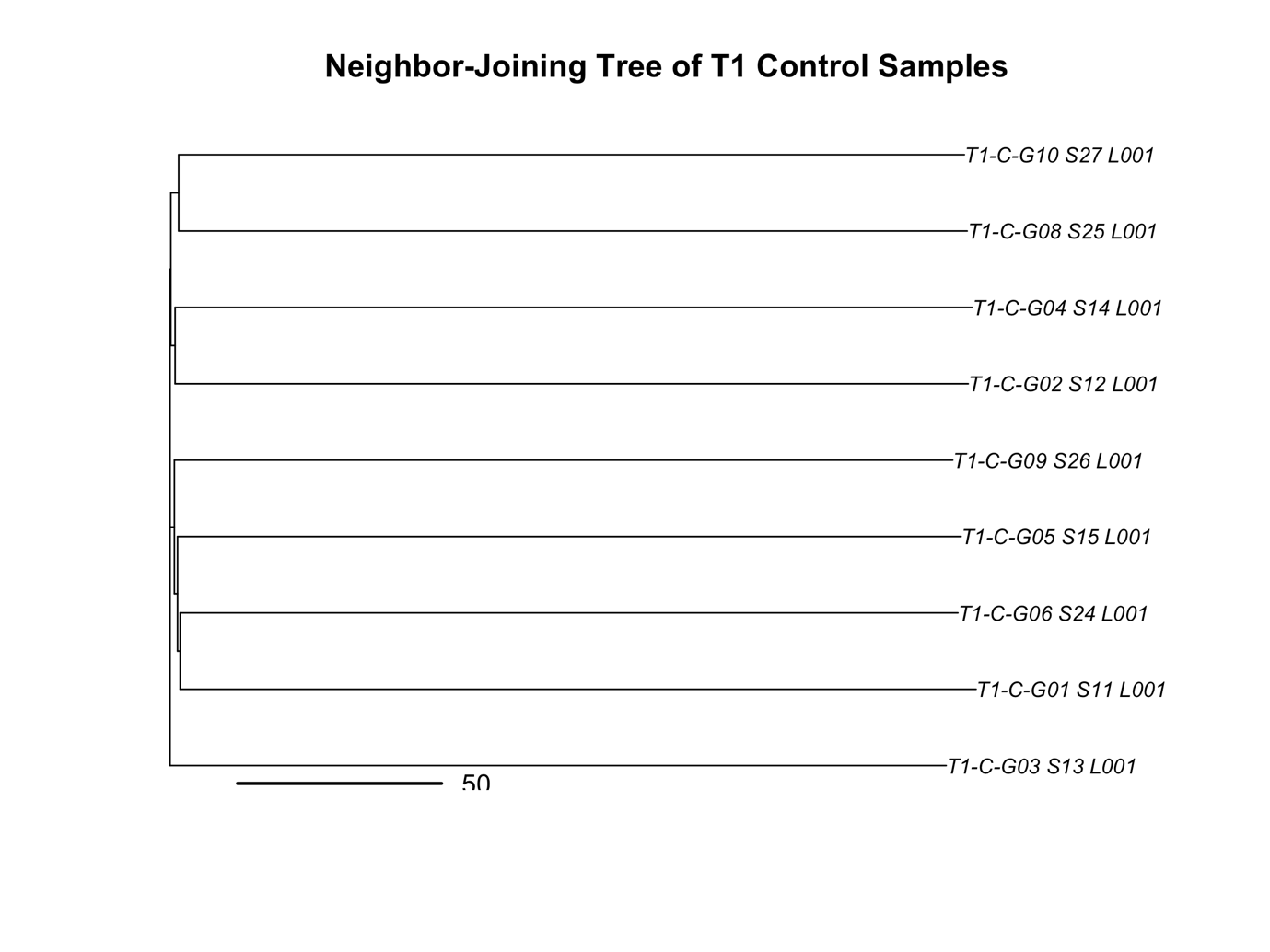

Figure S1. Neighbor-Joining tree of coral genotypes from the T1 control group based on SNP genotype distances. The tree was constructed using Euclidean distances calculated from filtered and imputed SNP data, and visualised with a scale bar indicating genetic distance. Each tip represents an individual coral sample collected at Timepoint 1 (T1) under control (C) treatment conditions.
